# Prognostic value of expression of nuclear factor kappa-B/p65 in non-GCB DLBCL patients

**DOI:** 10.18632/oncotarget.14182

**Published:** 2016-12-26

**Authors:** Jing Wang, Min Zhou, Qi-Guo Zhang, Jingyan Xu, Tong Lin, Rong-Fu Zhou, Juan Li, Yong-Gong Yang, Bing Chen, Jian Ouyang

**Affiliations:** ^1^ Department of Hematology, The Affiliated DrumTower Hospital of Nanjing University Medical School, Nanjing 210008, Jiangsu, PR China

**Keywords:** diffuse large B-cell lymphoma, p65, NF-κB, international prognostic index

## Abstract

**Purpose:**

We estimated the expression of nuclear factor kappa B/p65 in non-germinal center B-cell-like subtype diffuse large B-cell lymphoma, to investigate its relationship to clinicopathological features, and to further evaluate its prognostic value and clarify its impact on survival.

**Results:**

Among the 49 patients enrolled in this study, 14 (28.6%) had positive p65 expression. The negative p65 group had significantly better survival compared to the positive p65 group in terms of both the 3-year estimated OS (91.2% vs. 39.3%, p = 0.003) and PFS (75.6% vs. 26.5%, p = 0.002). In patients with 4 or more risk factors, p65 was an independent prognostic factor of OS (HR 5.99, 95%CI=1.39-25.75, p=0.016) and PFS (HR 4.01, 95%CI=1.15-14.00, p=0.029).

**Materials and Methods:**

The expression of the NF-κB/p65 protein was deteremined by immunohistochemistry in 49 non-GCB DLBCL. Survival was assessed by the Kaplan–Meier method and Cox multivariate analysis. The median patient follow-up period was 24 months.

**Conclusions:**

The expression of NF-κB/p65 has prognostic value in high risk non-GCB DLBCL, and it is a suitable target for the development of new therapies.

## INTRODUCTION

Diffuse large B-cell lymphoma (DLBCL) is the most common non-Hodgkin lymphoma (NHL), accounting for 25-35% of all new non-Hodgkin lymphoma diagnoses made globally each year [1]. Although gene expression profiling (GEP) has identified distinct DLBCL subtypes based on the differential expression of genes involved in B-cell development [2–8], it is not practical to perform GEP at most clinical institutions. Several groups have attempted to use immunohistochemistry (IHC) to distinguish between the germinal center B-cell (GCB) and non-germinal center B-cell (non-GCB) subtypes of DLBCL. Hans et al. used IHC for CD10, bcl-6 and MUM-1 to subdivide DLBCL into GCB and non-GCB subtypes [9]. The Hans classifier showed a concordance with the GEP gold standard of 71-93%[9–11]. The standard frontline treatment for patients with DLBCL, established over a decade ago, is rituximab, cyclophosphamide, doxorubicin, vincristine, and prednisone (R-CHOP) immunochemotherapy [12]. IHC-based cell of origin (COO) is a strong prognostic biomarker for identifying patient groups with substantially different outcomes following R-CHOP [7, 8, 13–16]. IHC algorithm had strong prognostic power matching that of GEP in DLBCL patients [16]. The use of an IHC algorithm has been widely adopted diagnostically, and has been incorporated into the British Committee on Standards in Haematology (BCSH) guidelines for lymphoma.

The nuclear factor kappa B (NF-κB) family of transcription factors control genes implicated in B-cell activation, proliferation and resistance to apoptosis [17]. In normal B cells, NF-κB activity was critical for B-cell development and survival [18]. NF-κB was constitutively activated in non-GCB DLBCL [19–20] and may be associated with drug resistance and a poorer prognosis [21]. Dimers of NF-κB family members (p50/105, p52/100, p65/RelA, p65/RelB and p65/c-Rel) mediated NF-κB-dependent transcriptional activities, and these molecules were regulated by members of the IκB family of inhibitors, which binded to NF-κB dimers and retained them in the cytoplasm [22]. Many downstream genes, which were involved in the regulation of cell survival, cell cycle distribution, and apoptosis, were proved to be transactivated by the p65 subunit of NF-κB [23].

In this study of non-GCB DLBCL patients, the relationship between the expression of NF-κB/p65 protein and clinicopathological parameters, and the prognostic value of NF-κB/p65 protein expression were explored.

## RESULTS

### Patients

The median age of the patients was 59 years (range 18–77 years), with 23 women and 26 men. The main characteristics of the patients at diagnosis are listed in Table 1. The distribution of patients according to the biological marker-adjusted International Prognostic Index (B-IPI) [24, 25] (n = 49) was as follows: low risk, 2 cases (4%); low/intermediate risk, 18 cases (37%); high/intermediate risk, 12 cases (24%); and high risk, 17 cases (35%).

**Table 1:** Clinical features of patients

Patient Characteristic	Value
Number	49
Median age, y (range)	59(18-77)
Male sex (%)	26(53)
**IPI factors**	
Age > 60 y (%)	23(47)
ECOG ≥ 2 (%)	10(20)
Elevated LDH (%)	25(51)
More than 1 extranodal site (%)	36(73)
Stage III/IV (%)	34(69)
**IPI**	
Low risk (%)	5(10)
Low / intermediate (%)	14(29)
High / intermediate (%)	19(39)
High risk (%)	11(22)
**Tumor characteristics**	
High MYC expression (%)	25(51)
High BCL-2 expression (%)	29(59)
MYC and BCL-2 coexpression (%)	18(37)
**B-IPI**	
Low risk (%)	2(4)
Low / intermediate (%)	18(37)
High / intermediate (%)	12(24)
High risk (%)	17(35)

### Clinicopathological significance of p65 expression

The associations between patient characteristics and p65 expression are shown in Table 2. Of the 49 patients examined, 14 (28.6%) had positive p65 expression. Positive expression of p65 protein showed a trend of correlation with patient age (p = 0.055), but not with other clinicopathologic factors, including sex, disease stage, LDH level, B symptom, and MYC and BCL-2 expression levels. Of the 14 tumor tissues with positive p65 expression, 6 were positive for MYC and 9 were positive for BCL-2.

**Table 2:** The relationship between p65 expressions and clinicopathologic parameters

Clinicopathologic parameters	N	P65		*χ*^2^	*p*^a^
		+(n=14)	-(n=35)		
**Sex**					
Male	26	7	19	0.074	1.000
Female	23	7	16		
**Age,years**					
<60	26	4	22	4.720	0.055
>60	23	10	13		
**Stage**					
I-II	15	2	13	2.460	0.174
III-IV	34	12	22		
**B symptom**					
Yes	23	8	15	0.819	0.528
No	26	6	20		
**ECOG**					
0-1	39	9	30	2.827	0.124
2-4	10	5	5		
**LDH**					
Normal	24	6	18	0.294	0.754
High	25	8	17		
**MYC**					
Low	24	8	16	0.523	0.538
High	25	6	19		
**BCL-2**					
Low	20	5	15	0.211	0.754
High	29	9	20		
**MYC and BCL-2 coexpression**					
Yes	18	5	13	0.009	1.000
No	31	9	22		
**IPI Scores**					
0-2	19	3	16	2.484	0.194
3-5	30	11	19		
**B-IPI Scores**					
0-3	20	4	16	1.217	0.344
4-7	29	10	19		

### Survival analysis

**Figure 1:**
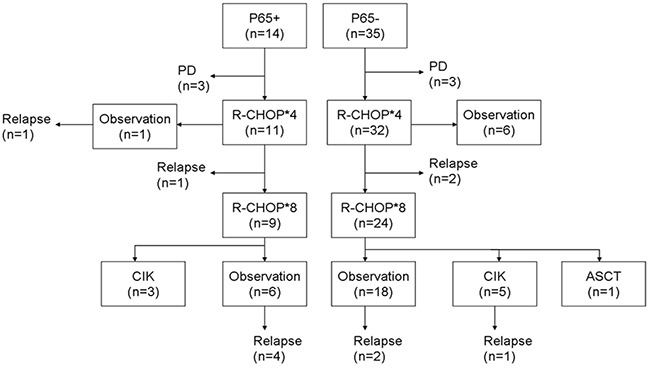
Summary of the treatments and responses ASCT autologous stem cell transplantation, CIK cytokine-induced killer cells, PD progressive disease.

**Figure 2:**
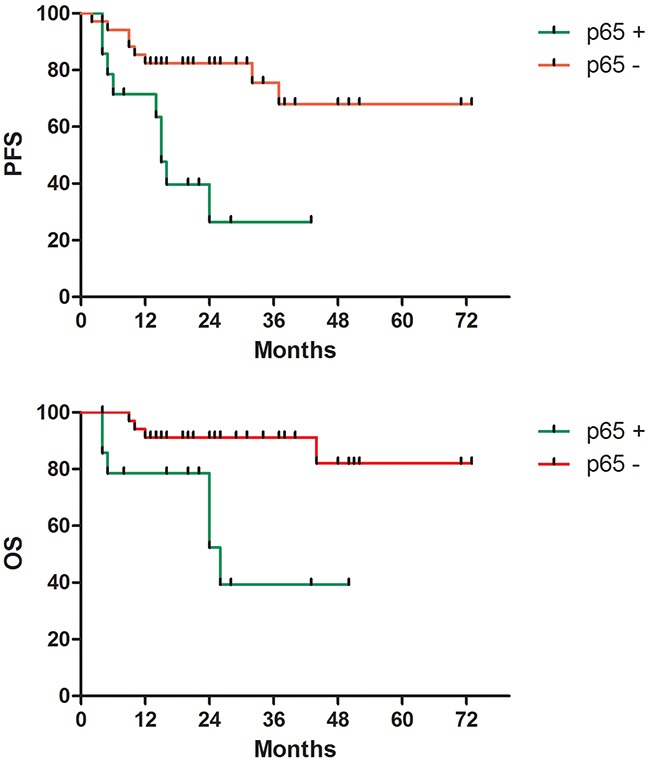
Survival according to p65 protein expression **A**. Progression-free survival (p=0.002). **B**. Overall survival (p=0.003).

**Figure 3:**
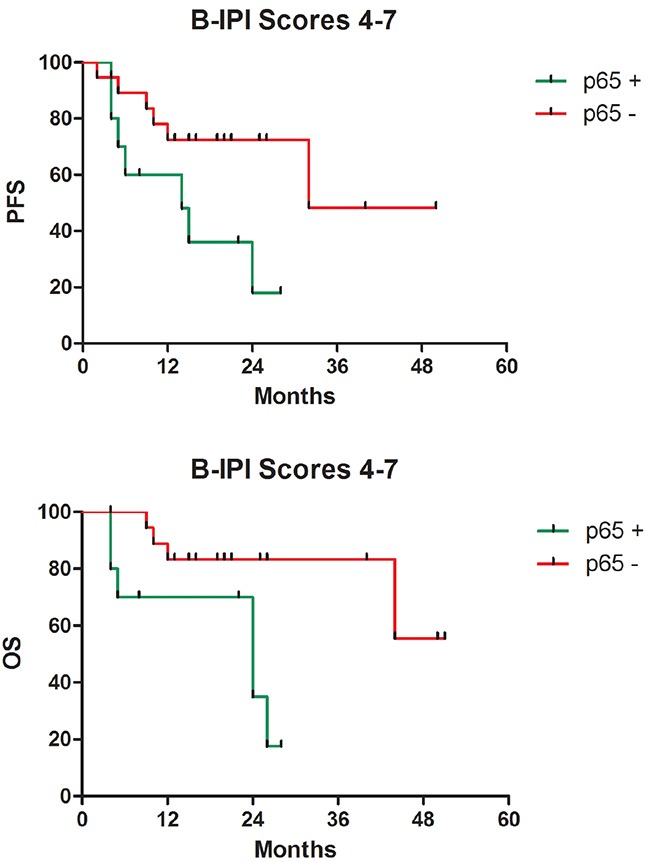
Survival according to p65 protein expression in patients with 4 or more than 4 risk factors **A**. Progression-free survival (p=0.016). **B**. Overall survival (p=0.029).

Six patients showed disease progression, whereas 11 patients relapsed during the treatment with R-CHOP (Figure 1). For all patients, the 3-year estimated OS was 76.7%, and the PFS was 61.6%. The negative p65 group showed a significantly better survival compared to the positive p65 group in terms of both 3-year estimated OS (91.2% vs. 39.3%, p = 0.003) and PFS (75.6% vs. 26.5%, p = 0.002) (Figure 2). The data for the 49 patients was examined by Cox multivariate analysis, and the B-IPI was proved to be an independent predictor of survival (Table 3). For patients with 4 or more risk factors, the Cox multivariate analysis showed that p65 was an independent prognostic factor of OS (HR 5.99, 95%CI=1.39-25.75, p=0.016) and PFS (HR 4.01, 95%CI=1.15-14.00, p=0.029) (Figure 3).

**Table 3:** Survival analysis in patients with non-GCB DLBCL

	Parameter	Univariate analysis		Multivariate analysis	
		HR(95%CI)	*p*	HR(95%CI)	*P*
**PFS**	IPI	3.75(1.17-12.04)	0.026	1.59(0.91-2.77)	0.854
	B-IPI	3.92(1.23-12.48)	0.021	2.26(1.13-3.88)	0.003
	BCL-2	4.17(1.19-14.66)	0.026	2.14(0.49-9.34)	0.306
	MYC and BCL-2	2.27(0.87-5.94)	0.093	1.00(0.32-3.16)	0.595
	P65	4.11(1.55-10.91)	0.004	4.85(1.74-13.51)	0.003
**OS**	IPI	5.99(2.09-17.8)	0.001	3.62(0.97-10.69)	0.796
	B-IPI	9.15(1.64-50.93)	0.011	8.13(1.47-44.76)	0.016
	BCL-2	6.39(0.81-50.49)	0.079	1.23(0.95-14.31)	0.872
	MYC and BCL-2	4.08(1.05-15.79)	0.042	2.27(0.46-11.15)	0.974
	P65	4.50(1.51-19.92)	0.009	4.02(1.00-16.19)	0.050

## DISCUSSION

DLBCL is an aggressive subtype of non-Hodgkin’s lymphoma (NHL) with diverse clinical and molecular characteristics. Currently, the International Prognostic Index is the most successful clinical model for predicting DLBCL outcome. Many efforts have been made to improve this model’s discriminatory capabilities [24–30]. Several prognostic models have been built for DLBCL. We have showed that B-IPI, a biomarker-based prognostic model, is a reliable and clinically applicable tool for predicting DLBCL prognosis [25, 26].

GEP has shown that a specific subgroup of DLBCL called activated B-cell-like (ABC) DLBCL has constitutive activation of the NF-κB system [31]. Furthermore, abrogation of NF-κB activity *in vitro* causes rapid cell death in non-GCB lymphoma cells [31, 32]. Characterization of DLBCLs with NF-κB activation is of great importance, as it may aid in identifying lymphomas for which NF-κB can be targeted for therapeutic intervention. Theoretically, treatment with the proteasome inhibitor bortezomib, a potent inhibitor of the transcription and nuclear translocation of NF-κB [33, 34], may overcome the negative prognosis associated with non-GCB disease in comparison to GCB disease in clinical practice [35, 36]. However, a phase 2 randomized study showed that substituting bortezomib for vincristine in the standard R-CHOP regimen did not significantly improve prognosis in patients with previously untreated, IHC-confirmed non-GCB DLBCL [37]. Subgroup analyses demonstrated no statistically significant differences in baseline IPI score between patients treated with VR-CAP and RCHOP. It is possible that the lack of difference in efficacy between VR-CAP and RCHOP therapies is because NF-κB activity only serves as a prognostic factor for the high risk non-GCB subtype of DLBCL (B-IPI scores ≥4), which was observed in our study. Bortezomib may be sufficiently active in that lymphoma subtype, which is characterized by constitutive NF-κB activation and shows resistance to current therapeutic modalities.

The classical NF-κB activation pathway involves dimerization of p50 and p65 or c-Rel. Those dimers are held inactive in the cytoplasm by specific inhibitors known as the inhibitor of κB (IκB) protein [22]. IκB kinase phosphorylates the NF-κB-bound IκBs, which targets the IκBs for ubiquitin-dependent degradation and allows the p65/p50 complex to translocate to the nucleus and initiate transcription of target genes [22]. The p65/p50 pathway has been shown to promote inflammation, cell proliferation, and cell survival through the production of several inhibitors of apoptotic signaling and to contribute to angiogenesis, tumor promotion and metastasis [22]. Nuclear expression of NF-κB has been reported in various tumor types and is considered a sign of NF-κB activation.

Our previous study showed that addition of MYC and BCL-2 to the IPI may allow its outcome prediction to be more clinically relevant. MYC overexpression sensitizes cells to NF-κB-mediated apoptosis, and a persistent lack of NF-κB signaling is a prerequisite for MYC-mediated tumorigenesis [38]. The NF-κB activation pathway was found to be constitutively active, inducing the expression of anti-apoptotic genes, such as BCL-2 and inhibiting the action of pro-apoptotic (Bax and Bak) proteins to promote cell survival [39]. Offner et al recognized that evaluation of MYC and BCL-2 expression in a phase 2 randomized study may provide further insight into the results of previous studies; however, such analyses were precluded by the lack of sample availability [37]. Here, a positive correlation between MYC, BCL-2 and p65 was not found. This was a retrospective study with a small number of patients; therefore, the patient cohort may not be representative of the general population of non-GCB DLBCL patients.

Our study showed that p65 is an independent predictor of survival in high risk non-GCB DLBCL. Further exploration is required to better clarify the role of the NF-κB pathway and, in particular, determine the subgroup of non-GCB DLBCL for which the pathway will serve as a valuable therapeutic target. When that information is gained, the combined therapeutic approach obtained by blocking NF-κB and inducing the apoptotic response may result in the most favorable outcome possible for non-GCB DLBCL patients.

## MATERIALS AND METHODS

### Patients and treatments

We retrospectively studied 49 patients with de novo non-GCB DLBCL centrally confirmed by the Hans method [9] who were treated at the Nanjing Drum Tower Hospital from 2008 to 2014. The study cohort included 26 males and 23 females with an average age of 59 years (range 18–77 years). All patients enrolled were in treatment with 4 to 8 cycles of the R-CHOP regimen at 21-day intervals. The patients underwent (18)F-FDG PET/CT before starting on R-CHOP treatment, after cycle four of the treatment, and after completion of it. Some patients only received 4 cycles of the R-CHOP regimen because of economic reasons. This study was approved by the institutional review board, and all patients gave written informed consent.

### Immunohistochemistry

Formalin-fixed, paraffin-embedded tissue (FFPE) sections of 3 μm in thickness were placed on adhesive-coated slides. Heated antigen retrieval was performed by immersing the slides in EDTA buffer (pH 8.0) and heating them for 2 min in a steamer. An antibody against p65 (Beijing Zhongshan Golden Bridge Biotechnology Co. Ltd, dilution 1:100) was used in addition to an autostainer following the standard polymer method (Dako Autostainer Plus). MYC and BCL-2 staining was completed as we previously reported [24–26]. Immunohistochemistry was evaluated by 2 experienced hematopathologists using a multihead microscope. MYC immunostaining was scored as positive when 50% of the tumor cells had nuclear staining [24–26]. Expression of BCL-2 was evaluated with cytoplasmic staining, staining 30% was considered as positive [25, 26]. Expression of p65 was considered positive when 50% or more of the nuclei of the tumor cells were stained [40, 41].

### Statistical analysis

Overall survival (OS) was computed from the date of diagnosis to the date of either death or last documented follow-up. Progression-free survival (PFS) was calculated from the date of diagnosis to either progression or death from any cause. PFS and OS rates were estimated using the Kaplan-Meier method, and differences were assessed by the log-rank (Mantel-Cox) test. Cox multivariate analysis was performed to test the prognostic value of the factors. Hazard ratios (HR) and their 95% confidence intervals (CI) were also calculated. Associations between p65 expression and the clinical characteristics of the patients were described by the Chi-square test. Fisher’s exact test was also used when necessary. All data were statistically analyzed using a commercially available statistical software package (SPSS 19.0; IBM Corp., Armonk, NY, USA). All tests were bilateral, and *P*-value < 0.05 was considered statistically significant.

## Abbreviations

COO: cell of origin
DLBCL: diffuse large B-cell lymphoma
FFPE: formalin-fixed, paraffin-embedded tissue
GEP: gene expression profiling
GCB: germinal center B-cell
HR: hazard ratios
IHC: immunohistochemistry
PFS: progression-free survival
OS: overall survival.
